# The Excision of Hard Chancres

**Published:** 1878-06

**Authors:** 


THE EXCISION OF HARD CHANCRES.—*The Doctor:* Considerable success seems to have followed this practice, which has now been for some time followed by Professor Auspitz, of Vienna. From the Vierteljahrscrift fûr Derm. and Sypth., 1877, we learn that he has excised hard chancre in thirty-three cases, as first recommended by Hueter.in 1867. The results are: (1) In a large number of cases no further syphilitic symptoms appeared, although at the time of the operation there was almost invariable indolent enlargement of the inguinal glands. This fact Auspitz regards as a proof that the initial stlerosis is not a pathological result of a pre-existing general systematic infection, but a starting-point or original depot for the infective material by which syphilis is transmitted. (2) In those cases where no secondary induration appeared after excision in the seat of the former chancre, there were, as a rule, no further symptoms of syphilis. (3) In some cases excision was followed by a secondary induration and a general outbreak of cutaneous and other syphilitic phenomena; but here the probability is that either the whole of the original chancre was not removed, or that the disease had spread too far along the neighboring blood-vessels before excision was performed. (4) In four cases the hard chancre was preceded by a soft sore, and in none of these did general symptoms follow excision. (5) The operation can be recommended as a preservative measure against general infection where the induration has been of short duration, where no lymphatic glands are indurated but the inguinal glands, and no other syphilitic symptoms are to be detected, and where the chancre is favorably situated, aud can be properly dressed and attended to after the operation. (6) Further evidence is required to show whether excision exercises any influence on the duration or severity of the general syphilitic symptoms in those cases in which it fails to prevent their outbreak, but there are grounds for believing that it possibly may.—*Louisville Medical News.*

